# Placement of Outdoor Exercise Equipment and Physical Activity: A Quasi-Experimental Study in Two Parks in Southern California

**DOI:** 10.3390/ijerph17072605

**Published:** 2020-04-10

**Authors:** Mojgan Sami, Megan Smith, Oladele A. Ogunseitan

**Affiliations:** 1 Department of Public Health, College of Health and Human Development, California State University, Fullerton, 800 N. State College Blvd., #KHS-121, Fullerton, CA 92831, USA; 2 Department of Statistics, University of California, Irvine, CA 92697-1250, USA; megants@uci.edu; 3 Department of Population Health and Disease Prevention, University of California, Irvine, CA 92697-3957, USA; oladele.ogunseitan@uci.edu

**Keywords:** fitness zone, outdoor exercise equipment, SOPARC, physical activity, parks

## Abstract

To reduce the burden of chronic disease, the Centers for Disease Control and Prevention (CDC) funded the Orange County Partnerships to Improve Health (OC-PICH) project in Orange County, California. One of the strategies included adding outdoor exercise equipment (OEE) in two parks in Garden Grove and Anaheim. Using a quasi-experimental pre-post design, we evaluated park users’ physical activity levels before and after OEE installation using the System for Observing Play and Recreation in Communities (SOPARC). The OEE was installed along a walking path in Edison Park (Anaheim) and grouped within a single area (a “fitness zone”) in Garden Grove Park. In both parks, there were significantly greater odds of high-intensity physical activity overall after the installation—19% higher odds in Anaheim, and 23% higher odds in Garden Grove. However, the fitness zone area in Garden Grove had substantially higher odds of increased physical activity post-intervention (OR = 5.29, CI: 3.76–7.44, *p* < 0.001). While the increases in physical activity levels are consistent with past studies that link OEE to higher levels of physical activity among park users, our findings also suggest that the location and placement of equipment within a park may be an important factor to consider when improving park amenities for physical activity.

## 1. Introduction

The relationship between the built environment and physical activity is well-documented [1,2,3]. As a feature of the built environment, parks play an important role in activating communities [3,4]. The characteristics of parks that are most commonly associated with increased physical activity by park users include accessibility, perceived safety, quality of amenities, and level of park maintenance [3,5,6,7,8]. Regular park programming and community outreach are also correlated with an increased likelihood of park users engaging in physical activity [9,10]. Adding outdoor exercise equipment (OEE) has gained popularity as an inexpensive strategy for improving park amenities to increase the physical activity of park users [6,11,12]. While research shows that slightly more studies found increased physical activity levels with OEE in parks, the evidence base still has gaps that need investigation [11,13]. For example, no study has yet analyzed whether, or to what extent, the placement and location of OEE within a park matters [11,14,15]. To our knowledge, no studies have compared physical activity levels between parks with equipment placed along a walking path and parks with consolidated equipment placement in “fitness zones”. Our study in Orange County aims to bridge this gap.

The Orange County Partnerships to Improve Community Health (OC-PICH) recently identified Anaheim and Garden Grove in central Orange County, California, as facing higher rates of sedentary behavior than more-affluent cities in the county [16]. In collaboration with each city’s Parks and Recreation Department, OC-PICH identified Edison Park in Anaheim and Garden Grove Park in Garden Grove as ideal sites to test the hypothesis that installing new OEE will increase park users’ physical activity levels, and that fitness zones designated specifically for OEE will have greater impact on increasing physical activity. To test this hypothesis, Edison Park’s new equipment was placed along a walking path, while Garden Grove Park installed equipment in a concentrated fitness zone within the park. Using the System for Observing Play and Recreation in Communities (SOPARC), the research team implemented a quasi-experimental pre-post study design to evaluate the physical activity levels of park users before and after installation of the new OEE in both Edison Park and Garden Grove Park. Our initial study evaluated each park separately, while providing us with an opportunity for initial exploration of the connection between equipment placement in parks and physical activity levels.

## 2. Materials and Methods

### 2.1. Setting

Our quasi-experimental pre-post study took place in two recreational parks in Orange County, California—Edison Park in Anaheim and Garden Grove Park in Garden Grove.

#### 2.1.1. Edison Park

Edison Park is a 7.5-acre park in northeast Anaheim. Before the installation of the OEE (the “intervention”), the park already had a children’s playground, a football/soccer field, picnic tables, restrooms, 2 sand-based volleyball courts, and 5 pieces of OEE by Playworld^®^ (Playworld, Lewisburg, Pennsylvania, USA), which were adjacent to a walking path on one side of the park [17]. In September 2016, as part of the research project, the Anaheim Parks and Recreation Department worked with Greenfields Outdoor Fitness Inc. (https://gfoutdoorfitness.com/) to install 5 additional pieces of free-standing equipment in Edison Park and add pavement along new a walking path (total surface area of approximately one-quarter mile).

Because of the accessible size of the park, we were able to observe the entire park for physical activity levels before and after the installation. The 6 target areas we defined included (TA1) the walking path with existing OEE, (TA2) green space and walking path, (TA3) baseball field, (TA4) green space and soccer field, (TA5) green space, and (TA6) green space and walking path. The equipment additions in Edison Park included 3 pieces of OEE and a paved walking path in TA4, and 2 pieces of equipment and a paved walking path in TA5. All other target areas had no change.

#### 2.1.2. Garden Grove Park

Garden Grove Park is a 36-acre park with football/soccer fields, picnic shelters, a playground, a small skate spot, restrooms, and basketball and volleyball courts. Garden Grove’s Parks and Recreation Department worked with Greenfields Outdoor Fitness Inc. to install 15 pieces of OEE in a new fitness zone. Because of the relatively large size of the park, the observations did not include all 36 acres. The research team focused on 9 target areas that surrounded the fitness zone intervention: (TA1) green space, (TA2) green space and covered seating area, (TA3) community center surrounded by green space, (TA4) dog park, (TA5) basketball court and small skate park (“skate spot”), (TA6) green space, (TA7) green space, (TA8) paved parking, and (TA9) paved road/parking. The park added 15 pieces of OEE in a concentrated fitness zone inside TA1. All other target areas had no change.

### 2.2. System for Observing Play and Recreation in Communities (SOPARC)

Our quasi-experimental pre-post study relied on SOPARC, a direct observation protocol that uses a combination of ecological and momentary time-sampling for data collection and assessment of physical activity in community settings [18,19]. SOPARC protocols call for direct observations to be conducted in small, observable “target areas” and allow for multiple researchers to be in the field. The SOPARC tool allows researchers to collect basic demographic characteristics (i.e., age, gender, ethnicity) and record only three levels of physical activity (i.e., sedentary, walking, vigorous) in 15-min time intervals in each target area [18]. Accounting for the size of the parks and the areas under observation, we divided Edison Park into 6 target areas and Garden Grove Park into 9 target areas (see Section 2.1). Student research assistants from the University of California, Irvine received 20 hours of training on SOPARC by the lead author of this study to ensure inter-rater reliability during each interval of observation [20].

### 2.3. Institutional Review Board

The Institutional Review Board of the University of California, Irvine determined that this observational study in public parks required no review because it involved no direct interaction with human participants.

### 2.4. Data Collection

Data collection for this study was coordinated with the directors of each city’s Parks and Recreation Department and their respective procurement processes for equipment construction and installation. At Edison Park, we collected pre-intervention data in September 2015 and post-intervention data in January 2017. A total of 48 hours of observational data were collected in Edison Park. At Garden Grove Park, we collected pre-intervention data in April 2016 and post-intervention data in November 2016. A total of 72 h of observational data were collected in Garden Grove Park. (While season and weather are important considerations for outdoor data collection in most parts of the world, the cities of Garden Grove and Anaheim experience temperate climates throughout the year.) In both parks, each target area was monitored by a trained researcher for a period of 3 days pre-intervention and 3 days post-intervention, with data collected across four 1-hour intervals (7:00–8:00 a.m., 12:00–1:00 p.m., 3:30–4:30 p.m., and 6:00–7:00 p.m.) each day. A total of 32 trained researchers participated in field data collection, monitored by the first author of this study.

According to SOPARC guidelines, we divided each hour of monitoring into 15-min observation periods and recorded user activity continuously during each period [18]. One researcher was assigned to observe each target area for the duration of a 1-hour interval, which implies observations of four 15-min intervals. During those intervals, researchers recorded the gender, age group, and race/ethnicity of each user and categorized each user’s physical activity level as sedentary, walking, or vigorous. Equipment use (post-intervention) was coded as sedentary when the machines were not used as intended (e.g., used as seating rather than exercise) and as vigorous when each apparatus was used as intended. The smallest unit of measurement in the SOPARC data is 1 person-period, defined as a single park user who occupied a target area for all or part of a 15-min period. If a park user occupied the target area for longer than a single 15-min period, or if a park user left the target area and returned during a later period, the researcher recorded it as multiple unique person-periods [18]. Field inter-rater reliability checks were conducted to assess agreement on levels of activity, race/ethnicity, and age [19].

Park users’ recorded physical activity levels were subsequently converted into Metabolic Equivalent of Task (MET) scores, as recommended by the Centers for Disease Control and Prevention [21]. There were three possible MET scores: sedentary was assigned 1.5 METs, walking was assigned 3.0 METs, and vigorous exercise was assigned 6.0 METs [22]. We calculated a period-average MET score for each observation period in each target area by averaging the individual MET scores for all person-periods in the time frame. The period-average MET scores quantify the overall average level of user activity in each target area during each observation period. The following demographic characteristics were also recorded per person-period: gender (male or female); age group (child, teen, adult, senior); and race/ethnicity group (white, Hispanic, black, other).

### 2.5. Data Analysis

We calculated the distributions of demographic characteristics and then compared pre-intervention and post-intervention distributions using chi-square tests, overall and within each target area for each park.

While we had quantifiable demographic and physical activity level data, there were numerous unmeasured or unmeasurable differences between the two parks and their overall use by community members. We therefore analyzed physical activity data separately for each park. We used a proportional odds mixed-effects regression model to estimate the odds ratios for a higher physical activity level at post-intervention than at pre-intervention. The dependent variable, activity level, is ordered, with vigorous being the highest level, followed by walking, then sedentary. The unit of analysis in these models is the person-period. The proportional odds model was adjusted for age group, gender, race/ethnicity group, and day of the week (weekday or weekend day). To account for correlation among observations measured at the same time of day or in the same target area, we included random intercepts for time and target area. Similar proportional odds models stratified by target area were also fit to examine the association between activity level and intervention status (pre-intervention or post-intervention) in each target area.

Some target areas had distinct built-environment elements. The stratified models were designed to determine (a) in which of the individual target areas, if any, changes in physical activity occurred post-intervention, and (b) which areas of the park were the strongest drivers of the estimated changes in activity for the parks overall. We tabulated odds ratios (ORs) and 95% confidence intervals (CIs).

To assess the association between period-average MET score and park intervention status, we fit a linear mixed-effects regression model with period-average MET score as the outcome and period as the unit of analysis. The model was adjusted for day of week (weekday or weekend day). Random intercepts for time of day and target area were included to account for correlation among measurements taken during the same time of day or within the same target area. We subsequently conducted a stratified analysis of the association between period-average MET and intervention status within each target area. The resulting regression coefficients for intervention status yielded the estimated differences in mean period-average MET score (either overall or by target area), which compared the intensity of physical activity post-intervention with pre-intervention park use. The statistical models used here were described with additional detail in a study of park users’ physical activity levels in Eastgate Park in Garden Grove, California [12]. All reported *p* Values are derived from 2-tailed tests and assume a type I error rate of 0.05.

## 3. Results

### 3.1. Descriptive Characteristics of Park Users in Edison Park and Garden Grove Park

In Garden Grove Park, we recorded 6336 person-periods at pre-intervention and 5319 person-periods at post-intervention. In Edison Park, we recorded 5628 person-periods at pre-intervention and 3238 at post-intervention. MET measurement data were collected for 288 pre-intervention and 288 post-intervention observation periods in Edison Park, and for 432 pre-intervention and 432 post-intervention observation periods in Garden Grove Park. Physical activity level measurements were at the person-period level; period-average MET measurements were at the period level.

In Edison Park, the distributions of all demographic characteristics (gender, age group, race/ethnicity group) differed at post-intervention from pre-intervention (*p* < 0.001 for each characteristic). In Garden Grove Park overall, there was strong evidence of differences between post-intervention and pre-intervention age groups (*p* < 0.001) and race/ethnicity groups (*p* < 0.001; see Table 1). More specifically, in Garden Grove Park’s TA1, the site of the fitness zone installation, the distributions of observed gender, age, and race/ethnicity of park users were all significantly different at post-intervention compared with pre-intervention (*p* < 0.001 for each characteristic).

### 3.2. Physical Activity Levels of Park Users Pre and Post-intervention in Edison and Garden Grove Parks

**Figure 1:**
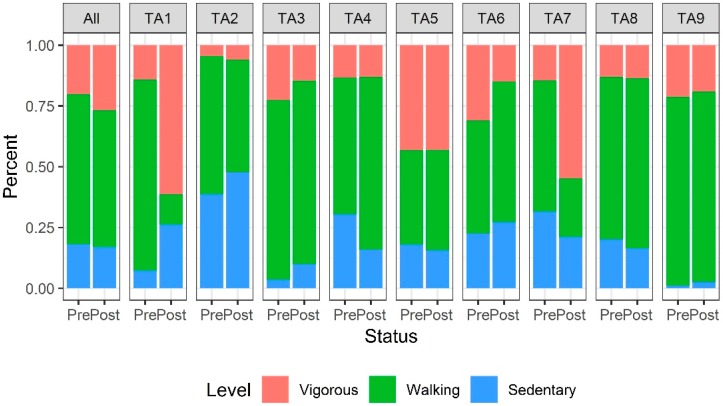
Distribution of physical activity levels in Garden Grove Park pre- and post-intervention. The numbers of observed person-periods pre-intervention and post-intervention overall and within each target area (TA) is listed in Table 2.

**Figure 2:**
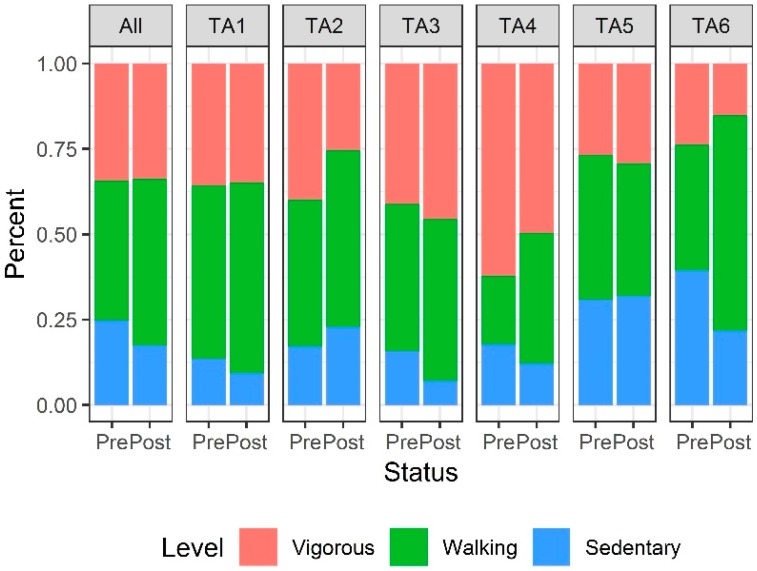
Distribution of physical activity levels in Edison Park pre- and post-intervention. The numbers of observed person-periods pre-intervention and post-intervention overall and within each TA is listed in Table 2.

The proportions of sedentary, walking, and vigorous activity levels observed among park users differed between pre-intervention and post-intervention both by target area and across each park overall (Figure 1 and Figure 2).

Overall, we estimated that post-intervention users in Edison Park had 19% higher odds of being classified in a more active category than pre-intervention users with similar demographic characteristics (OR = 1.19; 95% CI, 1.09–1.31; *p* < 0.001; Table 2).

Among the six target areas in Edison Park, post-intervention users had significantly higher odds of a higher activity level in TA1 (OR = 2.30; 95% CI, 1.74–3.04; *p* < 0.001), TA3 (OR = 1.83; 95% CI, 1.41–2.36; *p* < 0.001), and TA6 (OR = 1.32; 95% CI, 1.08–1.61; *p* = 0.007). Conversely, in TA2, post-intervention users had significantly lower odds of a higher activity level (OR = 0.74; 95% CI, 0.60–0.90; *p* = 0.003). Controlling for day of the week, the mean period-average MET score at post-intervention in Edison Park overall was an estimated 0.13 units higher (95% CI, −0.03–0.29; *p* = 0.10) than the mean period-average MET score pre-intervention (Table 3). This difference was not statistically significant, however, nor was there strong evidence to suggest a difference in post-intervention MET score within any of the specific target areas in Edison Park.

Post-intervention users in Garden Grove Park were estimated to have 23% higher odds of being classified in a more active category than pre-intervention users with similar demographic characteristics (OR = 1.23; 95% CI, 1.14–1.32; *p* < 0.001; Table 2). Among the nine target areas, post-intervention users had significantly higher odds of a higher activity level in TA1—the site of the fitness zone installation (OR = 5.29; 95% CI, 3.76–7.44; *p* < 0.001), TA4 (OR = 1.40; 95% CI, 1.12–1.74; *p* = 0.003), TA5 (OR = 1.34; 95% CI, 1.07–1.67; *p* = 0.011), and TA7 (OR = 3.61; 95% CI, 2.84–4.59; *p* < 0.001). Users had significantly lower odds of being classified at a higher activity level in TA3 (OR = 0.56; 95% CI, 0.41–0.77; *p* < 0.001), TA6 (OR = 0.72; 95% CI, 0.55–0.94; *p* = 0.016), and TA9 (OR = 0.77; 95% CI, 0.63–0.95; *p* = 0.016). Controlling for day of the week, the mean period-average MET score at post-intervention in Garden Grove Park overall was an estimated 0.08 units higher (95% CI, −0.02–0.19; *p* = 0.13) than the mean period-average MET score at pre-intervention (Table 3).

However, in TA1—the location of the fitness zone—the mean period-average MET score at post-intervention was 0.85 units higher (95% CI, 0.54–1.16; *p* < 0.001) than at pre-intervention. Meanwhile, three specific target areas in Garden Grove Park had significantly lower mean period-average MET scores at post-intervention than at pre-intervention: TA2 (estimated difference = −0.35; 95% CI −0.61 to −0.09; *p* = 0.009), TA3 (estimated difference = −0.32, 95% CI −0.57 to −0.07; *p* = 0.011), and TA6 (estimated difference = −0.37, 95% CI −0.67 to −0.08; *p* = 0.014).

## 4. Discussion

Physical inactivity has been repeatedly linked to a substantial burden of disease, including direct associations with health outcomes such as breast and colon cancers, cardiovascular disease, diabetes, and premature mortality [23]. The annual economic cost of physical inactivity has been estimated as $53.8 billion, about 60% of which is borne by the public sector and 25% by households [24]. Strategies to address this preventable disease burden have included research to map limitations to engaging in physical activities across age groups and investments in infrastructure to increase physical activity at the population level [25,26,27,28,29,30,31]. Our goal with the present study was to contribute to the national evidence base of best practices for incentivizing physical activity in urban populations that are experiencing disproportional burden of chronic diseases, with emphasis on public recreational parks. Our hypothesis was that the presence, quantity, and location of OEE in recreational parks will influence intentional physical activity by park users. 

This study of distributed OEE in Edison Park and OEE concentrated in a fitness zone in Garden Grove Park found a 19% (Edison) to 23% (Garden Grove) increase in users’ activity levels across the parks. This finding is consistent with our earlier study in Garden Grove’s Eastgate Park, where users had 40% higher odds of more vigorous activity after the installation of OEE [12]. The new data are also consistent with numerous prior studies of the role of public parks in promoting physical activity for different subpopulations [32,33,34]. Our results advance the state of increasing evidence that OEE is positively correlated with increased activity levels in parks, through data suggesting that this influence occurs regardless of whether the equipment is placed in a specific area of the park or spread throughout the park along a walking path. Perhaps our most interesting finding, however, was the significantly higher odds of engaging in higher levels of physical activity in Garden Grove’s fitness zone (529%) compared with any other target area in either park. Although our analysis did not specifically compare one park with the other, this finding suggests that equipment placement within parks merits further evaluation as more than a design element alone. Instead, our findings suggest that the placement or location of OEE should be considered as a variable in future research into the effects of such equipment installations on physical activity.

Although our findings support a strong association between equipment installation and higher physical activity levels among park users overall, we cannot draw conclusions on causality without considering potential confounding factors, such as funding requirements, timing of equipment installation, season of year, calendar time, and other environmental or population demographic changes in Garden Grove and Anaheim that occurred between the pre-intervention and post-intervention observations. The question of seasonality is one potential limitation of this study. Although it might be expected that data collected in fall or winter months could differ from data collected in spring or summer months simply due to weather limitations, we note that the Orange County, California, region experiences temperate climates throughout the year. Another limitation is that the researchers collecting data could not be blinded to intervention status, so conscious or unconscious bias in the classification of physical activity levels is possible, as is misclassification of park users’ demographic characteristics (e.g., ethnicity), despite the extensive training provided to all involved in data collection.

Furthermore, the original SOPARC tool for data collection only uses three levels of physical activity (sedentary, walking, vigorous) [18]. Therefore, we did not collect more detailed or nuanced information about the park users. In fact, these categories are the most granular information we have about each park user. This is a limitation of the tool itself, which was required for our study by the grant funder. As such, our current study is limited to reporting the findings from SOPARC, which is a validated tool used ubiquitously in physical activity and park research [15,18,19,20,33,34]. Ideally, we would have triangulated the data with other methods which collect more nuanced and detailed information on park users and not rely solely on direct observation, which is problematic on multiple levels, including the “observation” of race/ethnicity and age [35]. Collaborative empirical research projects, like the present study, befittingly work across federal agencies, academia, local government agencies, nonprofit organizations, equipment vendors, and timelines, and this collaborative work can prevent evaluation researchers from controlling all the variables needed for rigorous data collection methods. Such projects may have limitations in internal and external validity; however, they provide invaluable opportunities for mutual learning and success in addressing the health impacts of policy, systems, and environment (PSE) improvement projects [36].

## 5. Conclusions

Our study aimed to evaluate whether OEE would increase physical activity levels of park users. Overall, both of the California parks included in this study saw increased physical activity levels among park users after installing the new OEE. As part of our original hypothesis, we intuited that placement of equipment in a fitness zone will increase physical activity levels in the specific area of the park that received the intervention. Our findings suggest that placing exercise equipment in a centralized or consolidated “fitness zone” may indeed result in much higher impact on activity levels of park users than when the equipment is dispersed, such as along a walking path. This finding suggests that future research into improving park amenities for physical activity should include OEE placement or location as more than a design element, considering them as an important variable of investigation into the impact of physical activity.

## Figures and Tables

**Table 1:** Demographic characteristics of Park Users, pre- and post-intervention.

Characteristic	Garden Grove Park Target Area 1 ^a^		Garden Grove Park Overall		Edison Park Overall	
	Pre	Post	Pre	Post	Pre	Post
**Total no. of person-periods**	436	419	6336	5319	5628	3238
Sex						
% Male	64.9	49.6	66.4	64.8	60.5	68.7
% Female	35.1	50.4	33.6	35.2	39.5	31.3
χ^2^ (*p* Value)	19.75 (<0.001)		3.24 (0.072)		59.07 (<0.001)	
**Age group**						
% Child	3.9	19.3	9.8	10.2	31.6	20.2
% Teen	14.0	7.6	18.3	24.8	14.1	16.9
% Adult	64.0	32.2	59.9	45.5	42.8	53.7
% Senior	17.2	40.8	12.1	19.5	6.9	9.2
χ^2^ (*p* Value)	140.29 (<0.001)		273.76 (<0.001)		247.43 (<0.001)	
**Race/ethnicity**						
% White	19.5	3.6	15.1	11.7	3.2	5.1
% Hispanic	32.3	32.9	27.2	25.9	89.9	88.8
% Black	1.8	0.0	1.9	1.4	0.6	0.2
% Other	46.3	63.5	55.8	61.0	6.3	5.9
χ^2^ (*p* Value)	65.47 (<0.001)		44.75 (<0.001)		27.64 (<0.001)	

^a^ In Garden Grove Park, all new fitness equipment (i.e., the fitness zone) was located in target Area 1.

**Table 2:** Odds of higher physical activity levels post-intervention relative to pre-intervention.

Area	No. Observations Pre | Post	Activity Level Odds Ratio (95% Confidence Interval)	*p* Value
		**Edison Park**	
Overall	5628 | 3238	1.19 (1.09 to 1.31)	<0.001
TA1	839 | 374	2.30 (1.74 to 3.04)	<0.001
TA2	1153 | 720	0.74 (0.60 to 0.90)	0.003
TA3	621 | 549	1.83 (1.41 to 2.36)	<0.001
TA4	409 | 670	0.91 (0.69 to 1.21)	0.53
TA5	1322 | 411	1.22 (0.97 to 1.53)	0.083
TA6	1284 | 514	1.32 (1.08 to 1.61)	0.007
		**Garden Grove Park**	
Overall	6336 | 5319	1.23 (1.14 to 1.32)	<0.001
TA1 (fitness zone)	436 | 419	5.29 (3.76 to 7.44)	<0.001
TA2	446 | 333	0.75 (0.55 to 1.04)	0.083
TA3	609 | 431	0.56 (0.41 to 0.77)	<0.001
TA4	865 | 636	1.40 (1.12 to 1.74)	0.003
TA5	749 | 631	1.34 (1.07 to 1.67)	0.011
TA6	571 | 469	0.72 (0.55 to 0.94)	0.016
TA7	549 | 655	3.61 (2.84 to 4.59)	<0.001
TA8	1045 | 567	0.96 (0.76 to 1.22)	0.75
TA9	1066 | 1178	0.77 (0.63 to 0.95)	0.016

Abbreviation: TA = target area.

**Table 3:** Difference in mean period-average Metabolic Equivalent of Task (MET) score comparing post- to pre-intervention.

Area	No. Observations Pre | Post	Estimated Difference Pre- to Post-Intervention (95% Confidence Interval)	*p* Value
		**Edison Park**	
Overall	288 | 288	0.13 (−0.03 to 0.29)	0.10
TA1	48 | 48	0.23 (−0.15 to 0.62)	0.23
TA2	48 | 48	−0.31 (−0.62 to 0.01)	0.056
TA3	48 | 48	0.16 (−0.22 to 0.54)	0.39
TA4	48 | 48	0.28 (−0.20 to 0.75)	0.25
TA5	48 | 48	0.21 (−0.10 to 0.51)	0.19
TA6	48 | 48	0.26 (−0.11 to 0.64)	0.16
		**Garden Grove Park**	
Overall	432 | 432	0.08 (−0.02 to 0.19)	0.13
TA1 (fitness zone)	48 | 48	0.85 (0.54 to 1.16)	<0.001
TA2	48 | 48	−0.35 (−0.61 to –0.09)	0.009
TA3	48 | 48	−0.32 (−0.57 to –0.07)	0.011
TA4	48 | 48	0.04 (−0.16 to 0.24)	0.69
TA5	48 | 48	0.12 (−0.22 to 0.45)	0.49
TA6	48 | 48	−0.37 (−0.67 to –0.08)	0.014
TA7	48 | 48	0.68 (0.32 to 1.04)	<0.001
TA8	48 | 48	−0.06 (−0.31 to 0.18)	0.62
TA9	48 | 48	0.07 (−0.18 to 0.33)	0.58

Abbreviation: TA = target area.
